# Saccharin and aspartame excite rat retinal neurons

**DOI:** 10.3389/fopht.2023.1273575

**Published:** 2023-11-23

**Authors:** Jaeyoung Yang, Jason Myers, Malcolm M. Slaughter

**Affiliations:** Department of Physiology and Biophysics, Jacobs School of Medicine and Biomedical Sciences, The State University of New York at Buffalo, Buffalo, NY, United States

**Keywords:** TRPV1, sweet taste receptor, internal calcium, electroretinogram, glucose

## Abstract

Retinal sensitivity to a variety of artificial sweeteners was tested by monitoring changes in internal free calcium in isolated retinal neurons using Fluo3. Several ligands, including aspartame and saccharin elevated internal free calcium. The effects of these ligands were mediated by both ligand-gated membrane channels and G-protein coupled receptors. We explored the receptors responsible for this phenomenon. Surprisingly, mRNA for subunits of the sweet taste receptor dimer (T1R2 and T1R3) were found in retina. Interestingly, knockdown of T1R2 reduced the response to saccharin but not aspartame. But TRPV1 channel antagonists suppressed the responses to aspartame. The results indicate that artificial sweeteners can increase internal free calcium in the retinal neurons through multiple pathways. Furthermore, aspartame reduced the b-wave, but not the a-wave, of the electroretinogram, indicating disruption of communication between photoreceptors and second order neurons.

## Introduction

It is a pleasure to participate in a tribute to Steve Massey. He is a wonderful colleague and scientist. We were postdoctoral fellows together in the laboratory of the late Robert Miller. That was in the 1980’s, when the commercialization of aspartame was controversial and many people thought it might disrupt neural function, acting on glutamate receptors. Led by Jaeyoung Yang, we revisit the topic 40 years later.

Our understanding of sweet taste detection has been transformed by the discovery of the sweet taste receptor dimer, T1R2 and T1R3, in the tongue (1). The T1R2 protein is specific for sweet taste, while the T1R3 can also pair with the T1R1 to form the umami receptor. T1R2 knockout mice are deficient in tasting sweet chemicals such as sucrose, maltose, and saccharin (2). The sweet taste receptor is not only found in tongue, but also expressed in organs including pancreas, brain, and gut. In pancreatic cells, artificial sweeteners induce insulin secretion by activating T1R2+T1R3 receptors (3). Food deprivation or low glucose culture media can significantly increase T1R2 expression in brain and in a hypothalamic cell line, respectively (4). These studies indicate that sweet taste detection, mediated by T1R2+T1R3, occurs outside the gustatory system, including parts of the nervous system.

The retina expresses several family C G-protein coupled receptors, including the GABABR1 and GABABR2 subunits (5) and several of the metabotropic glutamate receptors (6–8). In exploring family C expression in retina, we found that T1R1, T1R2, and T1R3 mRNA were present. We also found that retinal neurons were activated by a variety of sweet tastants and that saccharin stimulated the T1R2/R3 receptor. A particularly unexpected result was the response to aspartame because rodent tongue is insensitive to this artificial sweetener (9). We tested several possible mechanisms by which rat T1R2/R3 might mediate the aspartame response but finally concluded that another receptor was involved. This turned out to be a TRPV1 channel. Interestingly, physiologically relevant levels of elevated glucose also activated this TRPV1 channel.

## Methods

### Retina dissociation and culture

All animal procedures were carried out in compliance with the National Institutes of Health Guide for the Care and Use of Laboratory Animals and were approved by the Institutional Animal Care and Use Committee of the University at Buffalo. Retinas were dissociated as described by Luo et al. (10) Immature (postnatal 10-20 days) Sprague-Dawley rats were anesthetized with halothane, decapitated, and the eyes were enucleated. The retinas were removed, washed in calcium- and magnesium-free Hank solution supplemented with 0.1 mM EDTA. The retinas were incubated in 0.2% pre-activated papain solution for 20 minutes at 37 **°**C. Individual cells were dissociated by gentle trituration and washing in calcium- and magnesium-free Hank solution. The dissociated cells were seeded onto 35 mm glass bottom culture dishes coated with poly-d-lysine. The retinal cells were maintained in Neurobasal A (Invitrogen-Gibco) supplemented with 2% B27 and 2% fetal bovine serum. Experiments were usually performed within 24 hours after dissociation. Rats were purchased from Harlan laboratory. TRPV1 knockout mice were purchased from the Jackson laboratory (B6.129S4-*Trpv1^tm1Jul^*/J). The genotyping of knockout mice was performed with the protocol suggested by Jackson laboratory.

### Electrophysiology

Whole cell recordings from retinal neurons and HEK293 cells were performed and analyzed using Axopatch 200B amplifier, PClamp software, and Igor Pro 5.03. Patch electrodes were fabricated from borosilicate glass (World Precision Instruments, Sarasota, FL, USA). Electrode resistances were 6-8MΩ. Pipettes contained (in mM): 130 potassium gluconate, 5 NaCl, 4 KCl, 10 HEPES, 2 EGTA, 4 ATP, and 0.4 Na-GTP (pH=7.2). Bath Hank solution contained (in mM): 146.5 NaCl, 1 NaHCO_3_, 0.3 Na_2_HPO_4_, 5 KCl, 0.3 KH_2_PO_4_, 0.5 MgSO_4_, 0.5 MgCl_2_, 5 HEPES-NaOH, 5 glucose, 1.25 CaCl_2_ (pH=7.4). In HEK293 cells expressing GIRK1/2, 25mM KCl extracellular solution was used in equimolar NaCl replacement. Test solutions were applied locally to the cells using an 8-channel, gravity-fed, manually operated microperfusion system. Almost all neurons had large voltage-gated sodium currents indicating that they were ganglion cells or amacrine cells.

### Calcium imaging

Cell permeant Fluo-3 AM (Invitrogen cat No F-23915) was used as a calcium indicator. The method for calcium dye loading was described by Otori et al. (11). Fluo-3-AM was dissolved in dimethyl sulfoxide to produce a 500 μM stock solution. Cells were incubated in culture media containing 5 μM indicator dye for 15 minutes at 37 **°**C and then washed for 15 minutes with superfused Hank solution at room temperature. All calcium imaging was performed at room temperature using an inverted microscope (Olympus IX81). The excitation illumination was 488 nm; emitted fluorescence was collected at wavelengths above 515 nm. Calcium fluorescence images, taken every 400ms, were analyzed with Slidebook 4.1 and Igor Pro 5.03. The pixel fluorescence intensities during stimulation were normalized with peak intensities induced by 50 mM KCl solution.

### T1R1 and T1R2 cloning

For full sequencing of T1R1 and T1R2 from retina, the 3’ Rapid Amplification of cDNA Ends (RACE) technique was performed with GeneRacer Kit (Invitrogen, Cat No. L1500-01). Reverse transcription was performed with Generacer oligo dT tagged with adaptor sequence. This reaction generated an adaptor tagged cDNA library. The 5’ primer for T1R1&T1R2 were designed against the 5’ untranslated region (UTR) of T1R1 & TR2. The full-length rat T1R1 and T1R2 were amplified with 5’ primer and gene racer 3’ primer.

### RT-PCR for whole retina tissue

Retinal tissue was isolated and homogenized with Trizol Reagent. The RNA was purified with TRIzol Reagent method (Invitrogen, Cat. No. 15596-018). The RNA was separated from other components by adding 0.2ml of chloroform and precipitated with 75% ethanol. The RNA precipitant was isolated by centrifugation and washed and dissolved in RNAse free water. Then 1 μg of total RNA was used for the RT reaction after the RNA concentration was measured by spectrophotometry.

The PCR for the cDNA library from each cell was performed using a Peltier thermal cycle system (MJ research, Waltham, MA). PCR was performed by adding 5 μl of template to a total volume of 50 μl mixture containing: 1X thermopol reaction buffer (20 mM Tris-HCl, 10 mM (NH_4_)_2_SO_4_, 10mM KCl, 2mM MgSO_4_, 0.1% Triton X-100, pH 8.8 at 25°C), 1 μl of each 50 μM sense and antisense primer, 1 μl of 10 mM dNTP (Invitrogen, Cat.No. 18427-013), 0.5 μl of Deep Vent Enzyme (200U/ml)(New England Biolabs, Ipswich, MA), water to 50 μl of total volume. The conditions for DNA amplification included an initial denaturation step of 95°C for 4 minutes; 30 cycles of 1 minute at 95°C, 30 seconds at 58°C, 30 seconds at 72°C; and then 10 minutes at 72°C. Each 15μl of final product was electrophoresed in a 1.5% agarose gel stained with ethidium bromide (EtBr; 0.5 μg/ml). All the PCR products were confirmed by DNA sequencing.

All primers were designed to contain at least one intron in its product to distinguish genomic DNA from mRNA. The primers for PCRs were designed with the BiBiserv program offered from Bielefeld University Bioinformatics Server.

Primers for PCR

T1R1:

sense 5’-TCGTCAGAGCTGTGCTCAGCCATGCTG-3’

antisense 5’-GCACCTTTCCAGTGGATCAGGTAGTGC-3’

T1R2:

sense 5’-CATCCTCTACGGCTGTCACTTTGCTGTC-3’

antisense 5’-ACGCTGCAGAGAATGGCAGAGGAACACC-3’

T1R3:

sense 5’-TCAGAGCCTGTTCAACCCTGGCAGC-3’

antisense 5’-CTGCGCACCTGGCCATCTTTGCACTG-3’

### ERG

Whole rat retina was isolated and stored in a dark room for more than 30 minutes before recording. The whole retina was transferred onto filter paper and placed photoreceptor layer down onto a MEA (multielectroarray, Multichannel Systems, Reutlingen, Germany). Then the whole retina with the filter paper was pressed down by a u-shaped platinum wire weight to enhance the contact between MEA electrodes and the retinal tissue. The retina was kept at 37°C in Ames media (Sigma Aldrich, Cat No A1420) and continuously gassed with 95/5 oxygen/carbon dioxide. Light responses were triggered by a white light LED stimulation.

### The siRNA and real time PCR experiment

R28 cell lines were used for small interfering RNA experiments. The R28 cells were maintained in the same culture media used for HEK293 cells. The siRNA oligonucleotide targeting the C terminal region of T1R2 was synthesized (Invitrogen, CA, USA).

The siRNA targeted sequence: GATTGTATGCCAGGCACCTACCTCA

Negative control siRNA was purchased from Invitrogen (Cat no.46-2001). Lipofectamine 2000 (Invitrogen,Cat No. 11668019) was used as the siRNA carrier. RFP (red fluorescent protein) cDNA was cotransfected with siRNA to monitor the efficiency of transfection. T1R2 siRNA and negative control siRNA were transfected into R28 cells according to the manufacturer’s protocol. The siRNA effectiveness was measured using real time PCR and calcium imaging.

Total RNA of R28 cells was prepared 0 hr, 24 hr, and 48hr posttransfection with RNeasy Kit (Qiagen).The quality and concentration of total RNA was estimated by absorbance at 260 and 280nm using the Experion system (Biorad, CA, USA). After treating RNA with DNAse, total RNA (0.5 μg) was turned into cDNA with the Superscript III kit (Invitrogen Cat No.18080-051) following the manufacturer’s instructions.

Real time PCR was performed with the MyiQ2 two color real time PCR system (Biorad, CA, USA).

Real time PCR primers:

Control B actin primers:

sense 5’-TGCTGACAGGATGCAGAAGGAGA-3’

antisense 5’-CCGGACTCATCGTACTCCTGCT-3’

T1R2 primers:

sense 5’- GAGCCAGAATCCCTTCCAAAGCA-3’

antisense 5’-AACAAGGGTGGAGGCCCACA-3’

Each reaction contained: 10 μl SYBR® Green PCR Master Mix (Biorad, CA,USA), 1 μl forward primer 5 μM, 1 μl reverse primer 5 μM, 2.5 μl cDNA template and RNAse DNAse free water up to 20 μl. The standard program was: 95°C for 3 min, 40 cycles at 95°C for 10 s and 60°C for 20 sec. All reactions were carried out in duplicate in Bio-Rad 96-well plates. Relative quantification was performed using the comparative C(T) method.

The specificity of real time PCR was tested by analyzing melting curves generated after the PCR reaction by the following protocol: 15 s at 95°C, 15 s at 60°C and a 20 min slow ramp between 60 and 95°C. The statistics were calculated with iQ5 (Biorad, CA, USA) and REST 2009 (Qiagen, Munich, Germany) software.

The effect of siRNA was evaluated by counting drug responding R28 cells 72hr after transfection. Drug responses were evaluated using calcium imaging. 100μM ATP was applied to R28 cells at the end of each experiment to give a standard response (12, 13). The cells were counted as responding cells if the amplitude of the calcium signal induced by the ligand was bigger than 10% of the amplitude of 100nM ATP induced response (default setting of autopeak software). All calcium imaging data were recorded with Slidebook 4.1 and analyzed with autopeak detection using Igor Pro 5.03 software.

### Statistical analysis

Results are expressed as mean ± SD (standard deviation) or mean ± S.E. (standard error) as indicated. Data were analyzed by single factor ANOVA. In some data sets, the paired student t-test was used, and this was specifically mentioned. Statistical values were calculated by Microsoft Excel and p <0.05 was considered significant.

## Results

### Retinal neurons respond to sweet tastants

**Figure 1:**
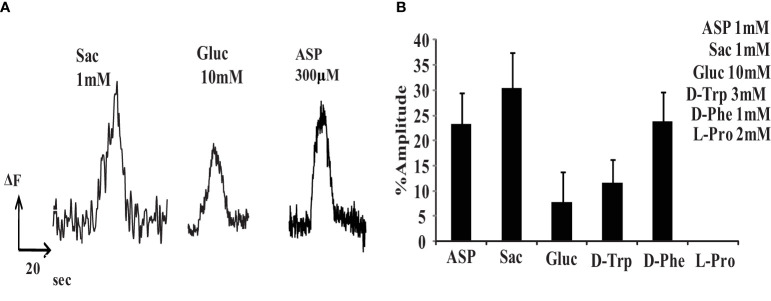
Various sweeteners can increase internal free calcium in isolated retinal neurons. **(A)** Examples of Fluo-3 fluorescent signals produced by saccharin, glucose and aspartame. **(B)** Relative Fluo-3 fluorescent signals in response to sweet tastants, normalized to the signal produced by 50mM KCl (% amplitude). 1mM aspartame (ASP) (23.3 ± 5.1%, n=13), 1mM saccharin (SAC) (30.3 ± 6.1%,n=9), 10mM glucose (GLUC) (7.7 ± 5%,n=11), 3mM D-tryptophan (D-TRP) (11.7 ± 3.6%,n=7), 1mM D-phenylalanine (D-Phe)(23.8 ± 4.7%,n=9), and 2mM L-proline (L-Pro)(0%,n=10).

Several sweet taste ligands stimulated retinal neurons. To examine this effect we measured changes in internal free calcium using Fluo3 in isolated rat retinal cells. Sweet tastants elevated internal free calcium and these responses were normalized to the elevation produced by 50 mM KCl. Several sweet tastants raised internal free calcium in dissociated retinal neurons: 1mM saccharin (30.3 ± 6.1%, n=9 cells), 10mM glucose (7.7 ± 5%, n=11 cells), 3mM D-tryptophan (11.7 ± 3.6%, n=7 cells) and 1mM D phenylalanine (23.8 ± 4.7%, n=9 cells) (**Figures 1A, B**). This was similar to the rat tongue’s sensitivity to these tastants (14, 15). However, rat retinal neurons also responded to 1 mM aspartame (23.3 ± 5.1%, n=13 cells). Although the human tongue responds to aspartame, rodents do not (16).

### T1R1, T1R2, and T1R3 are expressed in rat retina

**Figure 2:**
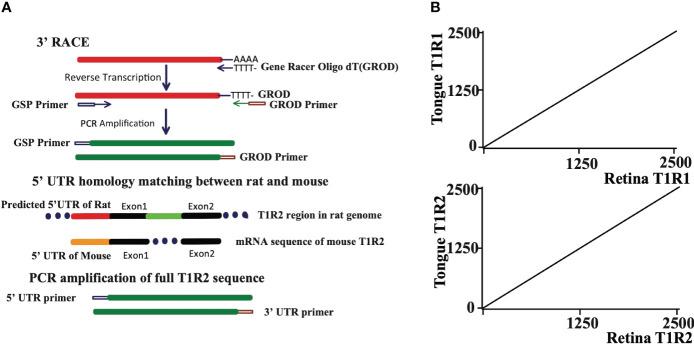
**(A)** The full sequences of T1R1 and T1R2 are the same in rat retina and tongue. The 3’ RACE (Rapid Amplification of cDNA Ends) technique was used to get 3’ terminal sequences of T1R1 and T1R2. 5’ UTR homology between rat and mouse was used to design 5’ UTR primer for T1R2. Known 5’ UTR sequence of T1R1 was used to design 5’ UTR primer for T1R1. The primer sequences are detailed in the text. **(B)** Dot matrix comparison of T1R1 and T1R2 sequences isolated from tongue and retina. Both sequences from retina were identical to those from tongue.

Since the sensitivity of our retinal cells to sweet tastants, with the exception of aspartame, was very similar to the sweet taste response profile in rat tongue we examined the expression of three taste receptor T1 genes in rat retina (T1R1, T1R2, and T1R3). Primer sequences were designed that spanned an intron to avoid amplification of genomic DNA. The 3’ RACE (Rapid Amplification of cDNA Ends) technique was employed to raise the detection levels of taste receptor isoforms. Gene Racer oligo DT, containing poly T to bind poly A tails of mRNA, was used in the 3’ RACE technique to pull out the 3’ ends of T1Rs in retina. The rat PCR primers for the 5’ ends were designed from the predicted homologous UTR (untranslated region) in the mouse genome. The full T1R1, T1R2 and T1R3 sequences were each determined to be identical to taste receptors in tongue (**Figures 2A, B**, results of T1R3 are not shown).

### Rat T1R2 is not responsible for the aspartame response

**Figure 3:**
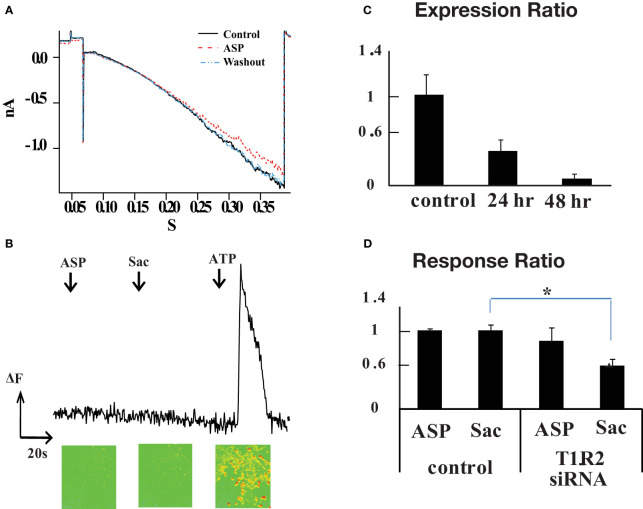
T1R2 is not involved in the aspartame response. **(A)** Absence of a GIRK current indicated that a functional sweet taste receptor cannot be made from cotransfection of T1R1 and T1R2 in HEK293 cells. G protein regulated inwardly rectifying K+ channel (Girk) 1 and 2 subunits were cotransfected with T1R1/T1R2 and GFP. GFP positive cells were chosen for the experiment. Ramp protocol from +10mV to -120mV was applied for 400 ms to transfected HEK293 cells (n=13). External Ringer solution contained 25mM KCl in equimolar replacement of NaCl to augment Girk current. 1 mM aspartame did not induce a significant Girk current. **(B)** Monitoring response of HEK293 cells after T1R1 and T1R2 cotransfection using fluo3 calcium imaging (n=214). T1R1 and T1R2 were cotransfected with modified G α15 protein, the final 5 amino acids of C terminal were mutated to Gi3 form. In contrast to ATP application, aspartame and saccharin did not evoke a calcium signal. **(C)** T1R2 mRNA levels decline with time after treatment with a siRNA against T1R2 Relative T1R2 mRNA expression in R28 cells was monitored with real time PCR for control cells and after 24 or 48 hour treatment with siRNA. Relative gene expression levels were calculated using the 2^−ΔΔ^*^C^*^T^ method. Beta actin gene was used as internal control. The y axis represents the expression ratio of T1R2 mRNA level of T1R2 siRNA transfected cells to control cells transfected with a scrambled siRNA. **(D)** T1R2 siRNA reduced the proportion of saccharin responding cells but aspartame responding cells were unaffected. After 72 hours, fluo3 calcium imaging was performed on siRNA treated R28 cells. The y axis is the response ratio (see text). There was a 45% reduction in the saccharin response of R28 cells after T1R2 siRNA treatment (*p =0.006, one-way ANOVA), but the change in aspartame response was statistically insignificant.

At this point we were confronted by a puzzle that rodent T1R2+T1R3 receptors do not respond to aspartame, yet aspartame produced strong calcium signals in retinal neurons. Since T1R1 is also expressed in retina, we tested whether rat T1R1 and T1R2, alone or in combination, could make a functional aspartame-sensitive receptor (14, 17). T1R1 and T1R2 cDNA derived from rat retina were transfected into HEK293 cells. Heteromeric G-protein-gated inwardly rectifying K^+^ channels (GIRK1/2) were cotransfected. GIRK channels can couple to various G protein coupled receptors such as acetylcholine, opioid, and dopamine receptors (18) and are activated by receptors that couple to Gαi/o and suppressed by receptors that couple to Gαq (16, 19, 20). To enhance the GIRK current the external Hank’s solution contained 25 mM KCl in replacement of NaCl. HEK293 cells were held at +10 mV, then voltage stepped or ramped to negative voltages before and during application of 1 mM aspartame. Negligible aspartame responses were observed, even at hyperpolarized potentials reaching -120mV (n=13 cells) (**Figure 3A**). Also, 1 mM saccharin and 30 mM D-glucose failed to affect the GIRK current in T1R1+T1R2 expressing cells. However, when GABA_B_ R1 and R2 subunits were cotransfected with GIRK1/2 and GFP, a GIRK current was generated by 10 μM baclofen, indicating that the co-transfection and response were viable (not shown).

A modified Gα is often used in reporter systems, wherein the final 5 amino acids at the C terminal in Gα15 are mutated to the Gαi3 form, Gα15i3 (17). The cDNA for the Gα15i3 was transfected into HEK293 cells along with T1R1 and T1R2 and GIRK cDNA constructs, but no aspartame or saccharin response was observed (n=214 cells) (**Figure 3B**).

### T1R2 knockdown in R28 retinal precursor cell line

In an alternative approach, the effect of knocking down the levels of T1R2 on the responses to aspartame and saccharin was tested. Ideally this would be done in isolated retinal neurons, but the retinal cell cultures did not survive the siRNA exposure. Consequently, the R28 retinal precursor cell line was used for siRNA experiments (21). The T1R2 mRNA expression was confirmed in the R28 cell line by RT-PCR. Both aspartame and saccharin (at 1 mM) raised intracellular calcium levels in R28 cells, tested using fluo3 calcium imaging.

A siRNA against T1R2 was designed and a scrambled sequence was used as a control. The RFP (red fluorescent protein) cDNA construct was cotransfected with T1R2 siRNA to monitor the transfection efficiency. RFP plus scrambled siRNA were cotransfected in control experiments. The mRNA levels of T1R2 in R28 cells were monitored 24 and 48 hours after T1R2 siRNA transfection using real time PCR. There was a 63 ± 12% reduction of T1R2 mRNA after 24 hours, and a 92 ± 5% reduction after 48 hours (n=3 dishes) (**Figure 3C**). The scrambled siRNA did not reduce the T1R2 levels. We did not measure changes in protein levels in response to siRNA.

After 72 hours, tastants were tested on transfected R28 cells using fluo3 calcium imaging. ATP (100 μM) was used to generate a control calcium signal. The cells not responding to 100 μM ATP were excluded from analysis. The cells responding to 100 μM ATP were divided into two groups: RFP positive and RFP negative cells. RFP negative cells were considered to be non-transfected controls with normal levels of T1R2/T1R3. If the amplitude of a tastant response was bigger than 10% of the 100 μM ATP response, that cell was counted as a sweet taste responding cell. The proportions of 1mM aspartame and 1mM saccharin responding cells in each group were calculated. The response ratio is the proportion of RFP positive cells (transfected with either target siRNA or scrabbled siRNA) that responded to aspartame or saccharin compared to the proportion of RFP negative cells (no transfection) that responded to these artificial sweeteners. The response ratios for aspartame and saccharin in the scrabbled vs. non-transfected groups were 1.075 ± 0.025 and 1.073 ± 0.008, respectively. Thus, in the scrambled siRNA experiments, approximately the same fraction of cells was responding to the artificial sweeteners in transfected and non-transfected cells. The response ratio for aspartame in the T1R2 siRNA test group was 0.93 ± 0.18 (p value=0.381, one-way ANOVA) and the response ratio of saccharin was 0.59 ± 0.09 (*p value=0.006) (control and siRNA each n=4 dishes, total number of cells=2368 and 2775 respectively). The siRNA produced a significant drop in the cells responding to saccharin, but there was little change in response to aspartame and this difference was not statistically significant (**Figure 3D**).

Thus, our results suggest that the rat T1R2 subunit is involved in the response to saccharin, but not aspartame. There was a 45% reduction in the R28 cells responding to saccharin, but the aspartame response was little changed by T1R2 siRNA treatment. This is consistent with rat behavioral studies showing only a weak gustatory response to aspartame (9) and with T1R2 expression studies indicating that aspartame activated the human sweet taste receptor but did not react with the rat sweet taste receptor. This leaves unresolved the receptor that mediates the rat retinal aspartame response. Interestingly, it also suggests that the effect of saccharin is not limited to stimulation of sweet taste receptors.

### Two mechanisms of aspartame-induced elevation of internal calcium

**Figure 4:**
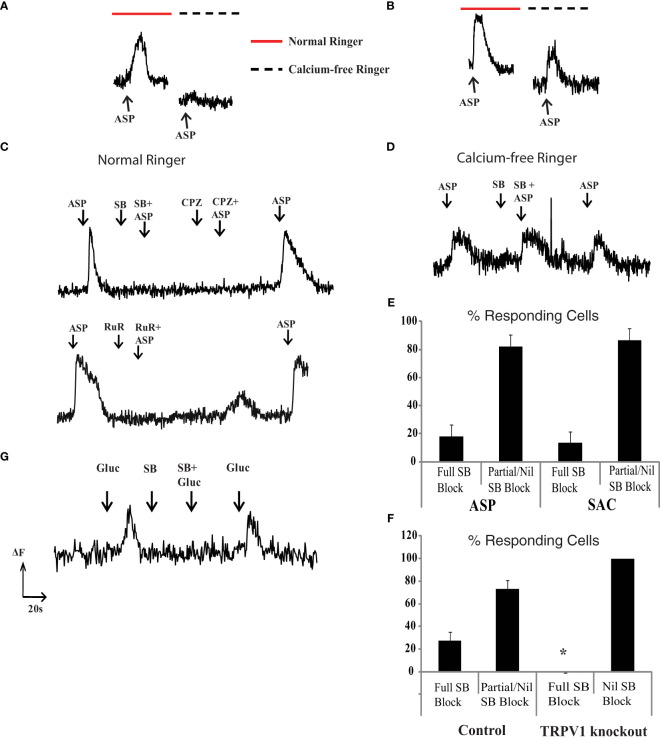
TRPV1 channel blockers suppress the Ca_out_ dependent aspartame, glucose and saccharin fluo-3 responses. **(A)** In some neurons the aspartame (300 μM) response was seen in the presence (red bar) but not the absence (dashed black line) of 1.25 mM external calcium. **(B)** In other neurons the response to aspartame persisted in the absence of Ca_out._ **(C)** The Ca_out_ dependent aspartame response was blocked by TRPV1 blockers SB (10μM SB366791), CPZ (10μM Capsazepine) and RuR (10μM Ruthenium Red). **(D)** Aspartame responses in Ca_out_ independent neurons were not blocked by 10μM SB366791. **(E)** Percentage of aspartame (ASP) and saccharin (SAC) responding neurons in which TRPV1 blocker (10μM SB366791) fully blocked responses as opposed to producing a partial or nil block. **(F)** TRPV1 knockout mice do not have TRPV1 blocker-sensitive aspartame responses. Percentage of aspartame (300 μM) responding cells that are completely vs. incompletely blocked by TRPV1 blocker (10μM SB366791) in wild type control and TRPV1 knockout mice (27 ± 8% block in control mice vs 0% block in TRPV1 KO mice, *p=0.0247 one way ANOVA). **(G)** 30mM Glucose induced intracellular calcium increases that were blocked by TRPV1 blocker (10μM SB366791, n=9).

The experiments indicate that aspartame, like saccharin and D-glucose, induce a rise in internal calcium in retinal neurons. However, there did not seem to be a direct link between T1R2 expression and the aspartame response. To explore this last point, external calcium was removed during aspartame application. In the absence of external calcium, aspartame responses disappeared in some rat retinal neurons (**Figure 4A**), but not in others (**Figure 4B**). This suggested that aspartame responses arose from two distinct pathways: 1) Ca_out_ dependent membrane channels and 2) Ca_out_ independent G-protein coupled receptor mediated internal release.

### TRPV1 antagonists block the Ca_out_ dependent aspartame response

The transient receptor potential vanilloid-1 (TRPV1) receptor can be activated by artificial sweeteners, as observed in heterologous expression systems and in dissociated primary sensory neurons (22). Furthermore, the TRPV1 receptor is expressed and elevates internal calcium in rat retinal ganglion cells (23). We tested whether the Ca_out_ dependent aspartame response arose from activation of TRPV1 channels that are calcium permeable. In cells where the aspartame response was Ca_out_ dependent, 10 μM SB366791, a selective TRPV1 blocker, completely eliminated the response. Two other TRPV1 blockers, 10μM capsazepine and 10 μM ruthenium red, had similar effects (**Figure 4C**). In contrast, cells in which the aspartame response was Ca_out_ independent were not blocked by SB366791 (n=18 cells) (**Figure 4D**). We found that 18 ± 8% of isolated retinal neurons possessed only Ca_out_ dependent aspartame (300μM) responses and these responses were completely blocked by 10 μM SB366791, a selective TRPV1 blocker (n=9 cells) (**Figure 4E**). The remaining aspartame responding neurons were at least partially Ca_out_ independent and the responses were not fully blocked by SB366791. Thus, TRPV1 channels accounted for the entire response in about one-fifth of aspartame-sensitive neurons. In the remaining responding neurons, the aspartame response was either exclusively from putative G-protein coupled receptors or from a combination of TRPV1 receptors and GPCRs (labelled “partial SB block” in **Figure 4E**).

To substantiate that the TRPV1 antagonists were indeed blocking TRPV1 receptors, the same experiments were repeated in TRPV1 knockout mice. The knockout mice should lack all forms of the TRPV1 receptor because it eliminates the transmembrane coding region of the gene. We confirmed this by genotyping in the knockout mice. The TRPV1 antagonists had no effect on aspartame responses in the knockout mice, although the antagonists were effective in age matched control mice (27 ± 8% block in control mice vs 0% block in TRPV1 KO mice, *p=0.0247 using single factor ANOVA, data from 3 KO and 3 control mice) (**Figure 4F**). This indicates that there is a set of rodent retinal neurons in which aspartame stimulates calcium influx through TRPV1 channels.

### Saccharin-induced responses in retinal neurons

The T1R2 siRNA results indicate that not all of the saccharin response in retinal neurons is due to activation of the canonical sweet taste receptor. Furthermore, saccharin-induced signals arose, like aspartame signals, from both Ca_out_ independent and Ca_out_ dependent pathways. The Ca_out_ dependent saccharin response was sensitive to TRPV1 blockers. In isolated retinal neurons, 10μM SB366791 completely eliminated saccharin responses in 13 ± 8% of cells (**Figure 4E**). TPRV1 antagonists had no effect or a partial block in the remaining 87 ± 8% of saccharin-sensitive neurons. Thus, saccharin responses involve a Ca_out_ dependent component produced by TRPV1 ionotropic receptors and a Ca_out_ independent component due a least partially to T1R2+T1R3 GPCRs.

### Glucose-induced responses in retinal neurons

In isolated retinal neurons we found that physiologically relevant increases in extracellular glucose (10-30 mM) evoked an elevation in free intracellular calcium like that of aspartame or saccharin. In 9 of 13 neurons responsive to 30 mM glucose, the response was completely blocked by 10μM SB366791 (as in **Figure 4G**), while in the remaining four cells the TRPV1 antagonist had no effect.

### Aspartame suppresses the B wave of the ERG in rat retina

**Figure 5:**
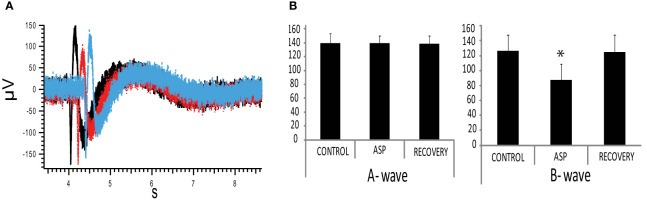
Aspartame reduction of ERG b-wave **(A)** ERG (electroretinogram) was measured in the isolated rat retina in response to a 1.5 s. white LED stimulation. 2mM aspartame (red trace) suppressed the positive-going B-wave. Black trace is control response and blue trace is recovery. Red and blue curves are shifted for viewing. **(B)** Summary of 2 mM aspartame effect on ERG: (Left) The A wave amplitude was not affected (Control -140 ± S.E. 4.5μV vs 2mM Aspartame 140 ± S.E. 3.5μV, p=0.481, paired t-test). (Right) The B wave was suppressed 30% in amplitude by 2mM aspartame (Control 126 ± S.E.5.6 vs 2mM Aspartame 88 ± S.E.2.1,* p=0.00001, paired t-test) (n=10).

To investigate the physiological effect of the aspartame-responding pathway in rat retina, we recorded the electroretinogram (ERG) triggered by white light stimulation. This is a light-evoked field potential in which the a-wave represents collective activity of photoreceptors while the b-wave represents the collective response of ON bipolar cells, neurons directly postsynaptic to the photoreceptors. When aspartame was applied to isolated, intact retina, the a-wave appeared unaltered (Control -140 ± S.E. 4.5μV vs 2mM aspartame 140 ± S.E. 3.5μV, p=0.481, paired t-test) while the b-wave of the ERG was suppressed by approximately 30% (Control 126 ± S.E.5.6 vs 2mM aspartame 88 ± S.E.2.1,* p=0.00001, paired t-test) (n=10, **Figures 5A, B**). This suggests that aspartame, activating either the TPRV1 receptor or a GPCR or both, modifies synaptic transmission at the first synapse in the retina.

## Discussion

Fortuitously, the rat’s gustatory insensitivity to aspartame facilitated an investigation of alternative transduction mechanisms for sweet tastants. The results demonstrate that artificial sweeteners activate multiple receptor systems in rat retina, including both channels and GPCRs. Two major findings are that the retina expresses: 1) a functional sweet taste T1R2+T1R3 GPCR, and 2) an ionotropic TRPV1 receptor stimulated by sweet tastants and glucose. Effectors of both systems converge on the control of internal free calcium.

### T1R2/T1R3

The sweet taste receptor in rat retina is identical to that in rat tongue (**Figure 2**) and not unexpectedly has a similar ligand sensitivity profile (**Figure 1**). We focused on the differences between artificial sweeteners: saccharin and aspartame. T1R2 knockdown partially reduced the saccharin response without effect on aspartame (**Figure 3**). This is consistent with the rat sweet taste receptor in tongue which is not aspartame sensitive. Even in primates, small variations in amino acids in the T1R2 binding pocket alter aspartame sensitivity (24). In the rat, differences in the “Venus flytrap” region of T1R2 account for this insensitivity. (25) (17, 26).

The function of T1R2+T1R3 in the nervous system is unclear (27). Since glucose is a main energy source for neurons, the possibility that the receptor monitors extracellular glucose is intriguing. The rise in T1R2 expression in hypothalamic cells due to reduced glucose suggests a link, but does not demonstrate that T1R2-containing receptors are directly activated by glucose (4). Furthermore, HEK-293 cells expressing rat T1R2 and T1R3 can be stimulated by sweet compounds such as sucrose and fructose but not glucose (1). The retinal GPCR response to saccharin and some D-amino acids matches well with the sweet taste system in the tongue, suggesting that it is responding to a similar ligand set. This receptor in retina may provide a means to detect carbohydrates and D-amino acids. For example, D-serine is important in NMDA receptor function and is detected by T1R2/T1R3 (28).

### TRPV1

The multimodal TRPV1 receptor was a good candidate for the rat retina aspartame response. TRPV1 receptors expressed in HEK293 cells can be activated by saccharin and aspartame (29) and TRPV1 channels are found in retina (23, 30, 31).

Based on the effects of TRPV1 knockout mice and TRPV1 antagonists, this retinal receptor is responsive to both saccharin and aspartame. TRPV1 plays a role in taste sensation in tongue, where it is proposed to complement umami and sweet taste responses and may account for the metallic taste of artificial sweeteners. (32). It is interesting, and perhaps important, that sweet tastants activate a combination of sweet taste and TPV1 receptors in both retina and tongue.

The aspartame TRPV1 signal may represent coding of extracellular glucose levels. Glucose produced a rise in internal calcium that was fully blocked by SB366791 in 70% of the neurons tested. The TRPV1 receptors have links with glucose metabolism. For example, insulin can sensitize TRPV1 channels in sensory neurons of the pancreas, while TRPV1 antagonists can increase insulin secretion and improve insulin resistance in diabetic mice (33). TRPV1 receptors are involved in weight control and glucose tolerance (34–39). N-oleoylethanolamide, an endogenous TRPV1 ligand, reduces food intake in wild type, but not in TRPV1 knockout mice (40). Multiple glucose-sensing mechanism exist in hypothalamic neurons, including the canonical mechanism found in pancreas (41, 42). The TRPV1 channel, producing an elevation of internal calcium, may complement the glucose response system as it does the sweet taste pathway.

### Electroretinogram

The b-wave of the electroretinogram (ERG) is reduced by aspartame, suggesting a depression of the photoreceptor synaptic signal. TRPV1 in monkey retina is associate with feedback inhibition from horizontal cells to photoreceptors (43), but there is no equivalent evidence in rodent, The effect of aspartame on light responses is a phenomenological tool but is not a clinical concern because aspartame is fully broken down to aspartate and phenylalanine in the gut and is not detectable in the blood.

### Detection by channels and GPCRs

For both the saccharin and aspartame responses, there was a component that required normal levels of extracellular calcium and a component that did not. This distinction is typical of the dichotomy between ionotropic and metabotropic receptor signaling, respectively. Consistent with that model, TRPV1 blockers inhibited only the calcium-dependent responses to both aspartame and saccharin. The metabotropic receptor in the saccharin pathway is the T1R2+T1R3 receptor. The Ca_out_ –independent, putative metabotropic pathway for aspartame was not identified, although some researchers have postulated that the cannabinoid receptor is the metabotropic correlate of TRPV1 because anandamide is an agonist for both receptors. Whether saccharin also activates this other GPCR is unclear, although behavioral studies raise that possibility (44).

## Data availability statement

The original contributions presented in the study are included in the article/supplementary material. Further inquiries can be directed to the corresponding author.

## Ethics statement

The animal study was approved by IACUC, University at Buffalo School of Medicine. The study was conducted in accordance with the local legislation and institutional requirements.

## Author contributions

JY: Writing – review & editing, Conceptualization, Data curation, Formal Analysis, Investigation, Methodology, Writing – original draft. JM: Investigation, Methodology, Software, Writing – review & editing. MS: Writing – review & editing, Supervision.
